# Relationship of Changes in Physical Fitness and Anthropometric Characteristics over One Season, Biological Maturity Status and Injury Risk in Elite Youth Ski Racers: A Prospective Study

**DOI:** 10.3390/ijerph17010364

**Published:** 2020-01-05

**Authors:** Lisa Steidl-Müller, Carolin Hildebrandt, Erich Müller, Christian Raschner

**Affiliations:** 1 Department of Sport Science, University of Innsbruck, 6020 Innsbruck, Austria; carolin.hildebrandt@uibk.ac.at (C.H.); Christian.raschner@uibk.ac.at (C.R.); 2 Department of Sport Science and Kinesiology, University of Salzburg, 5020 Salzburg, Austria; erich.mueller@sbg.ac.at

**Keywords:** injury risk, youth ski racing, change in fitness, change in anthropometrics, biological maturity status, talent development

## Abstract

Alpine ski racing is a sport with a high risk of injuries. In order to contribute to the longitudinal career development of young athletes, prevention measures should be elaborated. Therefore, the aim of the present study was to investigate prospectively the role of biological maturity status, and changes in anthropometric characteristics and physical fitness parameters over one season in elite youth ski racers younger than 15 years. Eighty-nine elite youth ski racers (39 females, 50 males), aged 10–14 years (mean age: 12.1 ± 1.3), were investigated. Anthropometric characteristics and physical fitness parameters were assessed prior and after the winter season; traumatic and overuse injuries were recorded over the 32 weeks. Binary logistic regression analyses (R² = 0.202–0.188) revealed that the biological maturity (Wald = 4.818; *p* = 0.028), and changes over the season in the jump agility test (Wald = 4.692; *p* = 0.03), in body height (Wald = 6.229; *p* = 0.013), and in leg length (Wald = 4.321; *p* = 0.038) represented significant injury risk factors. Athletes who could improve their jump agility performance more, had smaller changes in the anthropometric characteristics and who were closer to their peak height velocity were at a lower injury risk. In the context of injury prevention, regular neuromuscular training should be incorporated, and phases of rapid growth have to be considered.

## 1. Introduction

Alpine ski racing is a late specialization sport and the peak performance in elite alpine ski racing is mostly achieved between the ages of 26 to 28 years [1]. The specialization at an early age combined with a long athlete’s competitive life needs to be considered when implementing strategies to develop talents. Knowing this, skiing specific injuries are of particular interest especially in young athletes due to the fact that alpine ski racing is known to be a sport with a high risk of injuries [2]. At World Cup level, injury rates of more than 36 injuries/100 athletes were reported, of which more than 1/3 being severe and partly career ending [2,3]. The most affected body part is the knee at both World Cup level (35.6%) [4,5] and youth level with athletes younger than 15 years of age (36.5%) [6]. The rupture of the anterior cruciate ligament (ACL) is the most frequent reported diagnosis of all injuries at the World Cup level (13.6% of all injuries) [4], whereas at the youth level lower ACL rupture rates were recorded (e.g., 2 ACL ruptures among 67 youth ski racers aged 10–14 years over 2 seasons) [7].

Due to the high injury rates reported at both youth and elite levels, the investigation of injury risk factors in young athletes is crucial to contribute to the career development of the young athletes. Studies performed with ski racers younger than 15 years of age found the following significant injury risk factors. Athletes with weaker core flexion strength and weaker neuromuscular control were at a higher injury risk in general (10–14 year old athletes) [6] or had a higher risk of sustaining an ACL rupture (15–19 year old athletes) [8]. A decreased neuromuscular control of the knee was found to predict the risk of sustaining an ACL injury in female athletes [9]. Additionally, 10 to 14 year old youth ski racers with less pronounced anthropometric characteristics (weight, height, sitting height), and late maturing athletes were more vulnerable for more severe injuries (based on time-loss-definition) [6] and ski racers with high differences in unilateral isometric leg extension strength between the left and the right leg were at a higher risk of sustaining traumatic injuries in general, and most probably knee injuries [7]. Training load characteristics, such as training volume or intensity, among others, do not seem to influence the injury risk of youth ski racers [10]. However, most of the variability of the athletes in these studies remained unclear, because for example only 45%–47% of the variability could be explained by the regression analyses performed in the study by Müller et al. [6]. Nevertheless, when considering the multifactorial nature of injury causes in a changing outdoor environment, which decreases the chance of identifying statistically significant risk factors, as underlined by Spörri et al. [2], some risk factors could already be revealed, but a lot of work still has to be done, as for example identifying other athlete-related, equipment-related, or course-setting-related risk factors, elaborating possible prevention measures etc. [11]. Among these, modifiable risk factors such as changes in physical fitness parameters are of special interest for coaches. Even though the importance of a broad range of physical fitness on a high level, including strength, coordination, balance, endurance, and speed, are crucial in alpine ski racing, no study was performed concerning changes in physical fitness within a season in this sport, which was investigated in rugby [12,13] or soccer players [14], among others. Additionally, a study among soldiers revealed an association between declining aerobic fitness and increased utilization of medical resources [15]. However, to the author’s knowledge, the relationship between changes in physical fitness and occurrence of injuries within a season was not investigated in youth sport, yet. Additionally, even though it is known that growth-related factors contribute to overuse injuries [16] and that the adolescent growth spurt is a time of increased risk for sports injuries [17], changes in anthropometric characteristics over one season were not considered in research studies conducted with prepubescent or pubescent youth ski racers. Therefore, the aim of the present study was to investigate prospectively the role of biological maturity status, and changes in anthropometric characteristics, as well as changes in physical fitness parameters over one season in the context of injury risk identification in elite youth ski racers younger than 15 years of age. It was hypothesized that great changes in anthropometric characteristics would negatively affect the injury risk of youth ski racers. Additionally, it was hypothesized that performance improvements in fitness characteristics, especially in neuromuscular related parameters, would positively influence the injury risk of youth ski racers.

## 2. Materials and Methods

### 2.1. Study Design

The study was approved by the Institutional Review Board of the Department of Sport Science of the University of Innsbruck and the Board for Ethical Questions of the University of Innsbruck (#02/2014). A one-season prospective longitudinal study design was used to record traumatic and overuse injuries as well as changes in anthropometry and physical fitness parameters in a cohort of elite youth ski racers younger than 15 years of age. The biological maturity status, changes in anthropometric characteristics and physical fitness over one season as well as traumatic and overuse injuries were recorded. A sport specific internet-based database was developed and used in the present study; the database was reported by Müller et al. [6]. All tests were performed in the laboratory of the Department of Sport Science by experienced researchers. To ensure repeatability and to limit influences, the tests were conducted at the same time of day under standardized laboratory conditions, using the same measurement systems. The athletes had to wear standardized shoes of the laboratory and normal sporting clothes. The anthropometric characteristics and the physical fitness parameters were tested prior to the start of the skiing season (September) and at the end (May) of the season. The biological maturity status was assessed at the start of the study (September). Injuries were recorded from the beginning of the school in September until the end of the training in May; for data analyses each week of the study period was evaluated, except the vacations in December, February and April, thus in total, 32 training weeks over the whole season were analyzed.

### 2.2. Participants

In total, 89 elite youth ski racers (39 females, 50 males), aged 10 to 14 years (mean age: 12.1 ± 1.3 years), who were pupils of a ski boarding school and who all competed at national levels, were included in the study. The pupils were free of acute injury at the beginning of the study. The anthropometric characteristics of male and female athletes, as well as of the athletes of the three biological maturity status groups are presented in Table 1. All athletes, their parents and coaches were informed about the study aims, risks and benefits and the parents of the athletes provided their written informed consent.

### 2.3. Data Collection

#### 2.3.1. Physical Fitness Parameters

The current test battery represents the test battery for junior ski athletes as described by Raschner et al. [1], which was established in consultation with sports scientists, ski racing experts and coaches. The following parameters were included in the present analyses: Isometric core flexion and extension strength, isometric leg strength (left and right), postural stability (lateral and forward/backward direction), jump agility, counter movement jump, and drop jump. A detailed description of the testing procedures, measurement systems, number of attempts, and rest-retest-reliability values (ICC) can be found in the study of Raschner et al. [1,8].

#### 2.3.2. Anthropometric Characteristics and Biological Maturity Status

The anthropometric characteristics body height (0.5 cm; portable stadiometer SECA 217; SECA, Hamburg, Germany), body weight (1 N, Kistler force plate; Kistler Instrumente AG, Gommiswald, Switzerland; calculated to the nearest 0.1 kg), and sitting height (0.5 cm; portable stadiometer SECA 217; SECA, Hamburg, Germany; sitting height table) were assessed. The leg length as difference between body height and sitting height (0.5 cm) and the body mass index (BMI; 0.1 kg/m²) were calculated. The biological maturity status was investigated using the non-invasive method of calculating the age at peak height velocity (APHV) [18]. The gender-specific prediction equations included afore mentioned anthropometric parameters (except for BMI) as well as the calculated actual chronological age at the time of measurement. Based on this, the maturity offset, the time before or after individual peak height velocity (PHV), was then assessed in order to calculate the predicted APHV as difference between chronological age and maturity offset. A negative maturity offset indicated that the athlete is pre PHV, whereas a positive maturity offset indicates post PHV [18]. The validity of the APHV method was previously proven among youth ski racers of the same age as those who participated in the present study [19]. As performed by Sherar et al. [20] and other studies, the participants were then divided into three groups of maturity (late, normal and early maturing) based on the mean (M) ± standard deviation (SD) of the APHV of the total sample separated by gender. An athlete was classified as normal maturing if his/her APHV was within M ± SD; an early maturing athlete was categorized when his/her APHV was less than M-SD, and a late maturing athlete was identified when his/her APHV was higher than M + SD. Validity of this classification was also previously shown among youth ski racers and age-matched pupils [19].

#### 2.3.3. Injury Registration

Coaches of the ski boarding school recorded all relevant training data immediately after each skiing specific and athletic training session including among others duration, volume, intensity, contents, presence (or absence) of the athletes due to injury or illness etc. Exposure time was recorded for each athlete as the number of minutes of all training sessions of both skiing specific and athletic training. If an athlete was absent due to traumatic injury, overuse injury or illness, one member of the study team contacted the coaches, physiotherapists, and/or physicians of the ski boarding school to get detailed information. All injuries (traumatic and overuse) that occurred and that caused absence from training for at least one day, were registered. In case of injury that required medical attention, a detailed medical report was provided. Based on Brooks and Fuller [21] a traumatic injury was defined as an injury with a sudden onset based on time-loss definition. The type of traumatic injury and the affected body part were defined according to the injury surveillance consensus paper of the International Olympic Committee [22]. Injury severity was classified according to Fuller et al. [23]: An injury was classified as minimal with a time loss of 1–3 days, as mild (4–7 days), moderate (8–28 days), severe (>28 days), or career ending. Additionally, the mean injury severity of each athlete was calculated (total days of absence due to traumatic and overuse injury/total number of injuries). An overuse injury was defined as any physical complaint without a single identifiable event being responsible [24].

### 2.4. Statistical Analysis

Descriptive statistics are presented as the M ± SD for continuous variables and as frequency counts and percentages for categorical variables. The number of injuries was divided by the total number of athletes to calculate the injury rate per athlete. The incidence per 1000 h of training was calculated using the following formula: Number of injuries divided by the total number of hours of exposure of all athletes multiplied by 1000. The anthropometric and fitness characteristics tested at the beginning of the season were subtracted from the values tested at the end of the season to calculate changes in these parameters over the season. The normal distribution was tested using Kolmogorov–Smirnov tests. Two binary logistic regression analyses (backward LR method; dependent variable: Injury yes/no) were performed one for the changes in physical fitness parameters and one for the biological maturity status (APHV, maturity offset) and the changes in anthropometric characteristics. Traumatic and overuse injuries were combined for all analyses due to the small number of occurred overuse injuries. With respect to injury severity, multiple linear regression analyses with stepwise backward elimination were performed (dependent variable: Mean number of training days lost due to injury), one including the changes in physical fitness parameters and one including the biological maturity status (APHV, maturity offset) and the changes in anthropometric characteristics. Nagelkerke’s R² was calculated to determine the power of the model used. Independent t-tests were used to assess differences between injured and non-injured athletes with respect to the significant variables of the binary logistic regression analysis. The level of significance was set at *p* < 0.05. All calculations were performed using IBM SPSS 25.0 (IBM Corporation, Armonk, NY, USA).

## 3. Results

### 3.1. Changes in Physical Fitness over Season as Injury Risk Factor

The mean changes (± standard deviation) in the fitness parameters over the season are presented in Table 2 for the total sample and separated by athletes with and without injuries. The binary logistic regression analyses showed that the change in the jump agility test over the season was a significant predictor for injuries (Wald = 4.682; *p* = 0.03; R^2^ = 0.202). Athletes without injuries significantly increased their performance more than athletes with traumatic injuries (*p* = 0.03; T = 2.206). The other fitness parameter changes did not represent significant risk factors for injuries. When considering the severity of injuries, the total amount of days lost due to injury was considered; and the linear regression analyses did not reveal any significant parameter.

### 3.2. Biological Maturity Status and Changes in Anthropometric Characteristics as Injury Risk Factor

The mean changes (± standard deviation) in the anthropometric characteristics over the season, as well as the maturity offset are presented in Table 3 for the total sample and separated by athletes with and without injuries. The mean APHV of female athletes was 12.0 ± 0.4; for injured female athletes the APHV was 11.9 ± 0.3 and for non-injured female athletes the mean APHV was 12.1 ± 0.5. Male athletes showed a mean APHV of 13.7 ± 0.5; injured male athletes had in mean an APHV of 13.6 ± 0.7, whereas non-injured male athletes had a mean APHV of 13.7 ± 0.6. The binary logistic regression (R² = 0.188) revealed that changes in body height (Wald = 6.229; *p* = 0.013), changes in leg length (Wald = 4.321; *p* = 0.038) and the maturity offset (Wald = 4.818; *p* = 0.028) represent significant risk factors for injuries. However, no significant differences were found in these parameters between athletes with and without injuries. When considering the severity of injuries (total amount of days lost due to injuries), no significant risk factor was found with respect to changes in anthropometric characteristics and biological maturity parameters.

### 3.3. Injuries

A total of 53 medical problems were reported over the season: 41 traumatic injuries (0.46 injuries/athlete) and 12 overuse injuries (0.13/athlete). For further analyses, the traumatic and overuse injuries were considered combined due to the general small number of injuries (especially overuse injuries). An injury incidence of 1.4 injuries/1000 h of training was calculated. The 53 injuries were reported by 33 athletes (11 females, 22 males). Most injuries were mild or moderate (34.0% each), 17.0% were minimal and 15.0% were severe. None of the injuries was career ending.

## 4. Discussion

The present study was the first study that investigated the role of biological maturity status, changes in physical fitness parameters, as well as changes in anthropometric characteristics within one season in the context of injury risk identification in youth ski racers. Athletes who could improve their jump agility performance more over the season were at a lower injury risk. Additionally, the maturity offset, and changes in body height and leg length were significant injury risk factors; however injured athletes did not differ significantly from non-injured athletes in these parameters.

In the context of injury prevention in ski racing, high coordinative abilities play a crucial role. Modern ski racing techniques require a strong sense of stability because of the extensive inward leaning body angles [25,26]. For edging the skis with precision and for obtaining the correct feeling for carving, optimal sensorimotor abilities are required. Due to changes in speed, turning radii, terrain, snow and weather conditions while skiing, constant small adjustments are required [27]. As a consequence, well-developed balance abilities, but also other coordinative abilities are of utmost importance. This importance of high coordinative abilities in alpine ski racing is reflected also in the general recommendation of focusing on coordinative oriented strength training in youth ski racers instead of focusing on maximal strength training [28,29]. Another important aspect is that youth ski racers of the age category as in the present study, who primarily train slalom and giant slalom, do not need such a high amount of classical maximal strength training compared with older athletes who participate in speed disciplines, as well. Recent trends in athletic training in youth ski racing (e.g., slacklining, parkour, B-boying, and trampoline) follow these recommendations. It can be assumed that a good timing of muscle actions and the appropriate coordination affects jump coordination ability. The better the intra- and inter-muscular coordination of relevant muscle groups is developed, the better and more precise short-lasting movements can be performed. In alpine ski racing this aspect represents one of the most important in the context of performance enhancement and injury prevention. The continuous short-lasting stretch-shortening-cycles and the constant adaptations on the changing outdoor environment [25,26], require a very well-developed intra- and inter-muscular coordination, which is also true for the jump coordination test. The findings of the present study emphasize the importance of coordinative abilities in youth ski racing also in the context of injury prevention. The change in jump agility performance represented a significant injury risk factor (Wald = 4.682; *p* = 0.03). Injured athletes significantly differed from non-injured athletes (*p* = 0.03) in that way that non-injured athletes could improve their jump agility performance (+0.8 ± 0.8 s) more compared with the athletes who got injured during the season (+0.4 ± 0.5). The jump agility test was established for the test battery for junior ski athletes in consultation with sports scientists, ski racing experts and coaches [1], and can be associated with modern ski racing technique. The athletes have to jump bilaterally in forward, backward and lateral direction and during the lateral jumps the outer leg represents the dominant leg in pushing off the ground, which is the same in the modern ski racing technique. Additionally, during skiing, athletes continuously have to regulate their body’s center of gravity around the neutral position when compensating for external disturbances such as cuts, holes, different snow conditions, etc. The continuous regulation of the body’s center of gravity is also necessary during the jump agility test while jumping in forward–backward and lateral directions. Several studies have shown that deficient neuromuscular control can increase the risk of injuries in youth athletes [9,30]. However, to the author’s knowledge, no study assessed the changes in coordinative abilities over a season in the context of injury risk identification. In the study performed by Müller and colleagues [6], the jump agility performance did not represent a significant injury risk factor in youth ski racers. However, it can be assumed that these youth athletes all have a very high level of agility and performed well in the jump agility test, and thus, maybe no significant risk factor was found in the aforementioned study. When considering the performance development over the season, a difference could be revealed and the athletes who were able to improve their jump agility more were at a lower injury risk. However, it has to be mentioned that it could also be that injured athletes could improve their jump agility less due to the training loss because of the injury; a fact that cannot be neglected based on the present results. Nevertheless, the trainability of neuromuscular control is highest in preadolescent athletes [31] and neuromuscular training can have a preventative effect in reducing the risk of lower extremity injuries [32,33,34]. Therefore, neuromuscular training and testing including agility aspects should be incorporated regularly in the athletic-specific development of youth ski racers. Even though the athletes already represent high coordinative abilities, performance improvements positively affect the injury risk.

Taller, heavier and early maturing athletes have performance and selection advantages in youth alpine ski racing [35,36] and are at a lower injury risk or less vulnerable for severe injuries [37]. The adolescent growth spurt leads to anthropometric changes, and therefore, affects coordinative abilities [38]. Changes in anthropometric characteristics were not considered in the injury risk identification of youth ski racers, so far, even though Kemper et al. [39] showed that rapid growth rates and BMI increase might help to identify youth athletes at high injury risk. Not surprisingly, changes in body height (Wald = 6.229) and body weight (Wald = 4.321) were found to be significant injury risk factors in the present study, even though injured and non-injured athletes did not differ significantly from each other in these parameters. However, the higher the changes were, the greater the injury risk. Changes in BMI, however, did not represent a risk factor, as was found by Kemper et al. [39]. Based on these findings, injury prevention programs should include regular anthropometric measurements in order to identify phases of rapid growth, periods in which athletes seem to be at higher injury risk. Next to body height and body weight, the maturity offset as indicator of the biological maturity status, was proven to be a significant injury risk factor (Wald = 4.828). Even though injured and non-injured athletes did not significantly differ in their maturity offset from each other, the descriptive results indicate that non-injured athletes had a lower maturity offset, which means that they were closer to their PHV (in mean 0.6 ± 1.4 years before PHV) compared with the injured athletes who were in mean 1.1 ± 1.5 years before PHV. Previous studies showed that youth athletes are at a higher injury risk particularly between the year before peak height velocity (PHV) and the year of PHV [16,40], and that the adolescent growth spurt represents a time of increased risk for sports injuries [17], especially for overuse injuries [30]. Due to the general low number of injuries, and especially of overuse injuries, both types of injuries were considered combined in the regression analyses in the present study. Nevertheless, the present findings underline the necessity of considering the biological maturity status in injury prevention programs, especially for late maturing athletes.

## 5. Conclusions

Based on the findings of the present study, injury prevention measures in youth ski racing should focus on neuromuscular training sessions including agility aspects. Athletes who could improve their jump coordination performance more during the 32-weeks-season were at a significant lower risk for sustaining an injury. As the demands of the jump coordination test can be associated with those of modern ski racing technique, the importance of such training contents was emphasized. Additionally, when working with youth athletes prior to their individual peak growth spurt, phases of rapid growth have to be considered, as it was shown that greater changes in anthropometric characteristics were associated with a higher injury risk. Apart from that, athletes who were closer to their individual peak height velocity were at a lower injury risk. Based on these findings, training contents and intensities should be adjusted based on the individual development of the athletes.

## Figures and Tables

**Table 1:** Anthropometric characteristics of male and female athletes as well as separated by biological maturity status.

Anthropometric Characteristic	Gender		Biological Maturity Status		
	Male (*n* = 50)	Female (*n* = 39)	Early (*n* = 16)	Normal (*n* = 56)	Late (*n* = 17)
	M ± SD	M ± SD	M ± SD	M ± SD	M ± SD
Age (years)	12.2 ± 1.3	12.0 ± 1.3	11.6 ± 1.2	11.9 ± 1.3	13.0 ± 0.9
Height (cm)	152.5 ± 9.8	152.4 ± 10.1	155.8 ± 12.9	151.8 ± 9.6	151.7 ± 7.7
Weight (kg)	42.6 ± 8.9	43.5 ± 9.7	50.4 ± 10.5	41.3 ± 8.9	42.4 ± 6.0
BMI (kg/m²)	17.9 ± 2.0	18.5 ± 2.3	19.6 ± 2.5	17.7 ± 2.0	18.4 ± 1.8
Sitting height (cm)	79.0 ± 5.2	79.3 ± 5.1	81.9 ± 6.6	78.8 ± 5.0	78.1 ± 4.6
Age at peak height velocity (APHV) (years)	13.7 ± 0.5	12.0 ± 0.4	m (*n* = 11): 13.0 ± 0.2 f (*n* = 5): 11.4 ± 0.2	m (*n* = 29): 13.6 ± 0.5 f (*n* = 27): 12.0 ± 0.3	m (*n* = 11): 14.4 ± 0.1 f (*n* = 6): 12.6 ± 0.2

**Table 2:** Change in fitness parameters for total sample and athletes with and without injuries.

Fitness Parameters	Total (*n* = 89)	Injuries	
		Yes (*n* = 34)	No (*n* = 55)
**Change in jump agility (s)**	**0.7 ± 0.7**	0.4 ± 0.5 *	0.8 ± 0.8 *
Change in isometric leg strength right (N/kg)	−0.5 ± 1.9	−0.6 ± 1.8	−0.4 ± 1.9
Change in isometric leg strength left (N/kg)	−0.4 ± 2.1	−0.7 ± 2.2	−0.2 ± 2.1
Change in core flexion strength (N/kg)	0.4 ± 1.4	0.6 ± 1.3	0.2 ± 1.5
Change in core extension strength (N/kg)	0.1 ± 1.3	−0.1 ± 1.3	0.1 ± 1.4
Change in counter movement jump right (cm)	0.0 ± 2.3	−0.3 ± 2.8	0.1 ± 1.8
Change in counter movement jump left (cm)	0.2 ± 2.2	−0.1 ± 2.3	0.3 ± 2.1
Change in counter movement jump bilateral (cm)	0.9 ± 3.0	0.2 ± 2.8	1.2 ± 3.1
Change in reactive strength index (index)	0.0 ± 0.3	−0.1 ± 0.3	0.0 ± 0.3
Change in postural stability left/right (index)	−0.5 ± 0.8	−0.6 ± 0.8	−0.4 ± 0.8
Change in postural stability forward/backward (index)	−0.5 ± 1.2	−0.5 ± 1.3	−0.5 ± 1.1

Note: Change in fitness parameters: Positive value indicates better performance at end of season testing. * significant difference between injured and non-injured athletes (*p* = 0.03); bold value indicates significant risk factor.

**Table 3:** Biological maturity status and changes in anthropometric characteristics for total sample and athletes with and without injuries.

Anthropometric Characteristics	Total (*n* = 89)	Injuries	
		Yes (*n* = 33)	No (*n* = 56)
**Change in height (cm)**	**3.0 ± 1.5**	**2.8 ± 1.5**	**3.2 ± 1.5**
Change in weight (kg)	3.0 ± 2.0	2.9 ± 2.1	3.1 ± 1.9
Change in BMI (kg/m²)	0.5 ± 0.6	0.5 ± 0.6	0.5 ± 0.6
Change in sitting height (cm)	2.0 ± 1.2	1.9 ± 1.3	2.0 ± 1.1
**Change in leg length (cm)**	**1.4 ± 0.9**	**1.5 ± 0.9**	**1.3 ± 0.9**
**Maturity offset (yrs)**	**−0.8 ± 1.4**	**−1.1 ± 1.5**	**−0.6 ± 1.4**

Note: Change in anthropometric characteristics: Positive value indicates higher value at end of season testing; bold values indicate significant risk factors.
